# Ecological Mechanisms Underlying the Persistence of *Yersinia pestis* in the Natural Plague Foci of Kazakhstan: An Integrated Ecological Framework

**DOI:** 10.3390/pathogens15101091

**Published:** 2026-10-20

**Authors:** Ziyat Abdel, Tatyana Meka-Mechenko, Zauresh Zhumadilova, Raikhan Mussagalieva, Aigul Abdirassilova, Bolatbek Baitursyn, Anar Zarkymanova, Beck Abdeliyev, Nurbol Shaki, Svetlana Issaeva

**Affiliations:** M. Aikimbayev’s National Scientific Center for Especially Dangerous Infections, 14 Zhakhanger St., Almaty A35P0K3, Kazakhstan; ziyatabdel@gmail.com (Z.A.); tmekamechenko@gmail.com (T.M.-M.); zzbgdirect@nscedi.kz (Z.Z.); raikhansafar@gmail.com (R.M.); aigul.abdirassilova@gmail.com (A.A.); b.bola-1993@mail.ru (B.B.); azarkumanova@gmail.com (A.Z.); abdelbeck@gmail.com (B.A.); nurbol.shakiy@gmail.com (N.S.)

**Keywords:** *Yersinia pestis*, plague, natural foci, enzooty, enzootic cores, great gerbil, *Rhombomys opimus*, fleas, environmental persistence, biofilms, L-forms, persister cells, ecological model

## Abstract

Plague remains a significant natural-focal infection, yet the mechanisms underlying the long-term persistence of *Yersinia pestis* during inter-epizootic periods remain incompletely understood. This study integrated published evidence with epizootiological monitoring data from Kazakhstan (1990–2025) and detailed field and laboratory investigations conducted during 2021–2023 to develop an integrated ecological framework of plague persistence. Abiotic and biotic niches, reservoir hosts, flea vectors, and adaptive states of *Y. pestis* were evaluated according to the strength and type of supporting evidence. Long-term surveillance identified spatially recurrent enzootic cores and documented the continued presence of the great gerbil (*Rhombomys opimus*)–flea host–vector system across major desert plague territories. Field investigations covered approximately 120,000 km^2^ and included 4586 environmental and biological samples. Bacteriological examination yielded 24 viable *Y. pestis* isolates belonging to the Medievalis biovar, whereas real-time PCR detected *Y. pestis*-specific DNA in 88 of 4586 samples (1.92%), with the highest positivity observed in ectoparasite suspensions (66/868; 7.60%). Laboratory evidence was concentrated in five investigated foci containing recurrent enzootic core areas, while no positive findings were obtained in three comparison foci. These findings support an evidence-weighted ecological framework in which established host–vector transmission operates within spatially structured reservoir populations and heterogeneous environments, while additional environmental and bacterial persistence mechanisms remain plausible but require further experimental validation.

## 1. Introduction

Plague remains one of the best-studied yet conceptually challenging natural focal infectious diseases because of the ability of its etiological agent, *Y. pestis*, to persist in natural ecosystems for prolonged periods. Despite substantial advances in understanding plague biology and transmission, the mechanisms that allow *Y. pestis* populations to persist during prolonged inter-epizootic periods remain incompletely understood [1,2,3,4].

Long-term observations from natural plague foci in Central Asia, Kazakhstan, the Caspian region, Mongolia, China, Africa, and North America have demonstrated pronounced temporal variation in epizootic activity, with active periods alternating with prolonged intervals of apparent inactivity. In some plague foci, *Y. pestis* was not isolated for decades before epizootic activity reappeared [1,2,3]. Related evidence concerning plague persistence has been reported in Kazakhstan and Central Asia, as well as in experimental studies of environmental survival [4,5,6].

The classical epizootiological concept describes plague maintenance primarily through the rodent–flea–rodent transmission cycle [1,2]. Although this model explains the principal mechanism of epizootic transmission, it does not fully account for prolonged inter-epizootic intervals or the re-emergence of plague activity after extended periods without bacteriological detection. Experimental and field studies have therefore examined additional mechanisms that may contribute to pathogen persistence, including survival in soil and burrow substrates [7,8,9,10,11,12], intracellular persistence in free-living amoebae [13,14,15], biofilm formation and other adaptive physiological states [16,17,18,19,20], and associations with plant rhizospheres and other environmental substrates [21,22].

These observations have led to broader ecological concepts in which plague persistence may involve interactions among reservoir hosts, flea vectors, environmental substrates, soil biota, and adaptive states of *Y. pestis*. However, the strength of evidence differs among these proposed mechanisms: some are supported by the recovery of viable bacteria or experimental observations, whereas others are based on molecular detection or ecological associations. Consequently, environmental detection of *Y. pestis* DNA should not by itself be interpreted as evidence of viable bacterial persistence or of a confirmed environmental reservoir.

Kazakhstan, with its extensive and long-monitored natural plague foci, provides an opportunity to examine these mechanisms across multiple spatial and temporal scales. Accordingly, the present study integrates published evidence with long-term epizootiological surveillance, field and laboratory investigations, and spatial data from the natural plague foci of Kazakhstan. The aim was to characterize long-term host–vector and spatial patterns, evaluate field and laboratory evidence from selected natural foci, and integrate these findings with published data into an ecological framework of *Y. pestis* persistence, while distinguishing demonstrated observations from hypothesized persistence mechanisms.

## 2. Materials and Methods

The study integrated historical and long-term epizootiological surveillance data, original field and laboratory investigations, spatial analyses, and published evidence to examine ecological mechanisms associated with the persistence of *Y. pestis* in the natural plague foci of Kazakhstan. The design, temporal coverage, geographic scope, and analytical role of each evidence component are described below.

### 2.1. Study Design and Data Sources

This study used a descriptive and integrative design combining several complementary evidence streams that differed in temporal coverage, geographic scale, and analytical purpose. These included historical epizootiological records from Kazakhstan (1926–2022); long-term epizootiological surveillance data (1990–2025); original field and laboratory investigations conducted during 2021–2023; recent host–vector monitoring data from the national anti-plague surveillance system (2021–2025); and published national and international studies addressing ecological mechanisms of *Y. pestis* persistence.

The datasets were analyzed according to their respective temporal and methodological scope and were not treated as a single pooled dataset. Historical and surveillance records were used to characterize long-term spatial and epizootiological patterns, whereas the 2021–2023 project provided original environmental, bacteriological, serological, and molecular data from selected natural plague foci. Published studies were used for comparative evaluation of proposed persistence mechanisms. Integration of these evidence streams was performed at the interpretative stage to develop the ecological framework of *Y. pestis* persistence in the natural plague foci of Kazakhstan. The scope and analytical role of each evidence stream are summarized in Table 1.

### 2.2. Literature Survey and Comparative Ecological Analysis

The literature survey included classical studies of plague ecology [1,2], investigations conducted in Kazakhstan and other endemic regions [4,5,6,11,12,22,23], and experimental studies addressing environmental persistence and ecological adaptation of *Y. pestis* [7,10,13,14,15,16,18,20]. Published evidence was comparatively evaluated according to the environmental or biological substrate investigated, conditions associated with pathogen survival, reported duration and type of persistence evidence, proposed mechanisms, and potential ecological significance. Particular attention was given to distinguishing recovery of viable *Y. pestis* from molecular detection of bacterial DNA and from proposed or experimentally inferred persistence mechanisms.

### 2.3. Integrated Ecological Framework

Evidence from the literature, long-term epizootiological surveillance, field and laboratory investigations, and spatial analyses was integrated at the interpretative stage to develop an ecological framework of *Y. pestis* persistence. The framework considered interactions among reservoir hosts, flea vectors, environmental substrates, soil biota, and adaptive bacterial states. Evidence was interpreted according to its level of support, distinguishing demonstrated observations from potential or hypothesized persistence mechanisms.

### 2.4. Long-Term Epizootiological Monitoring (1990–2025)

Long-term epizootiological monitoring data from the natural plague foci of Kazakhstan were used to characterize spatial and temporal variation in the principal components of the host–vector system and plague activity. Monitoring covered the major natural plague territories of the country and included annual field surveys of rodent populations and their ectoparasites, with particular attention to the great gerbil (*R. opimus*), the principal reservoir host in desert plague foci. Field surveillance and epizootiological assessment were conducted according to established methodological approaches for epidemiological surveillance in natural plague foci [24].

The monitored parameters included the area occupied by great gerbil colonies, burrow occupancy, host population density, flea abundance, evidence of epizootic activity, and the extent of annually surveyed territories. Monitoring records were analyzed descriptively to characterize long-term ecological and spatial patterns rather than to test formal temporal trends.

### 2.5. Field Investigations and Sample Collection (2021–2023)

Original targeted field investigations were conducted during 2021–2023 in selected natural plague foci of Kazakhstan. The investigations included five autonomous plague foci containing recurrent enzootic core areas (Ural–Emba, North Aral, Aral–Karakum, Balkhash, and Ili Intermountain) and three comparison foci (Kyzylkum, Moyynkum, and Betpakdala). Detailed field surveys covered approximately 120,000 km^2^, with individual expedition routes ranging from approximately 300 to 1000 km. This targeted survey area was distinct from the substantially larger territory covered annually by the national anti-plague surveillance system (approximately 738,000–744,000 km^2^ during 2021–2025).

The study design included targeted investigation of territories associated with recurrent plague activity and comparative examination of selected territories in which no active plague epizootics had been recorded in recent years.

A total of 4586 environmental and biological samples were collected, comprising 1388 rodent-burrow soil samples, 725 burrow-substrate samples, 399 feeding-chamber content samples, 1168 sets of rodent biological samples, including liver, spleen, blood, and serum, 868 ectoparasite suspensions, and 38 rodent skeletal remains. Soil samples were collected from great gerbil (*R. opimus*) burrows at depths of 50, 100, 150, and 200 cm. Soil pH, temperature, moisture content, and salinity were assessed to characterize the physicochemical conditions of the burrow microenvironment. Field sampling was conducted together with epizootiological observations of host and flea-vector populations.

### 2.6. Laboratory Investigation of Field Samples

Field materials collected during the 2021–2023 investigations were examined using bacteriological, serological, and molecular methods routinely applied within the national plague surveillance system of Kazakhstan and described in the relevant methodological guidelines [25,26,27,28].

Bacteriological examination of appropriate biological and environmental materials was performed for the isolation and identification of viable *Y. pestis* according to established methodological guidelines [25,26]. Recovered cultures were identified using standard bacteriological and phenotypic criteria, followed by characterization of their principal biological properties and biovar determination. For bacteriological examination of rodents, liver, spleen, and blood samples were used. Rodent serum samples were used for F1-specific immunological testing and real-time PCR.

Serological investigations were performed according to established plague diagnostic procedures [26] using passive hemagglutination-based assays. F1-specific immunological testing included detection of the F1 capsular antigen and anti-F1 antibodies in rodent serum samples, whereas ectoparasite suspensions were examined for F1 antigen.

Molecular examination was performed by real-time PCR for the detection of *Y. pestis* DNA according to validated methodological recommendations [27,28]. DNA extraction was performed using the RealBest UniMag kit (Vector-Best JSC, Koltsovo, Novosibirsk Region, Russia) and the RIBO-prep kit (Central Research Institute of Epidemiology, Moscow, Russia). Molecular identification of *Y. pestis* isolates was performed using the Plague qPCR test system developed by the M. Aikimbayev National Scientific Center for Especially Dangerous Infections (Almaty, Kazakhstan) and the AmpliSens diagnostic system (Central Research Institute of Epidemiology, Moscow, Russia). Real-time PCR was applied to all 4586 field samples collected during the targeted investigations, comprising 1388 rodent-burrow soil samples, 725 burrow-substrate samples, 399 feeding-chamber content samples, 1168 rodent serum samples, 868 ectoparasite suspensions, and 38 rodent skeletal remains. PCR-positive results were interpreted specifically as molecular detection of *Y. pestis* DNA and not as evidence of bacterial viability.

Bacteriological, serological, and molecular findings were recorded separately according to diagnostic method, sample type, and study territory. All procedures involving viable *Y. pestis* were conducted within the institutional biosafety and containment framework established for work with particularly dangerous pathogens at the M. Aikimbayev National Scientific Center for Especially Dangerous Infections, in accordance with the applicable national biosafety requirements.

### 2.7. GIS and Spatial Analysis

Spatial analysis was performed to characterize the geographic organization and long-term distribution of plague activity within the natural plague foci of Kazakhstan. Historical epizootiological records, long-term national surveillance data, field observations, laboratory findings, and data on the spatial distribution of the principal reservoir host and flea vectors were integrated for comparative spatial analysis.

Enzootic cores were defined as geographically persistent areas characterized by recurrent plague activity over long-term observation periods. Their identification was based on the spatial recurrence of epizootiological evidence, including detection and isolation of *Y. pestis*, together with the long-term presence and spatial stability of the principal host–vector system. Historical and contemporary epizootiological records were compared spatially to identify territories in which plague activity repeatedly occurred within the same geographic areas.

Spatial mapping and analysis were performed using ArcGIS 10.8 (Esri, Redlands, CA, USA). GIS-based mapping was used to visualize the spatial distribution of plague activity and to delineate the boundaries of the identified enzootic cores. The spatial analysis identified four major clusters of recurrent enzootic core areas, which were further compared with the distribution of reservoir hosts and flea vectors and with relevant landscape and environmental characteristics.

The resulting spatial data were analyzed descriptively and integrated with field, laboratory, and ecological evidence to evaluate the role of geographically stable enzootic cores within the proposed ecological framework of *Y. pestis* persistence.

### 2.8. Comparative and Statistical Analysis

The study was primarily descriptive and comparative in design. Quantitative data were summarized using absolute numbers, proportions, percentages, ranges, and annual or territory-specific indicators, as appropriate. Comparisons were performed among natural plague foci, sample types, observation periods, and selected ecological characteristics.

Long-term surveillance data on great gerbil distribution, burrow occupancy, population density, flea abundance, and epizootiological activity were evaluated descriptively to characterize temporal and spatial variation. Annual values were compared across observation periods without formal statistical trend modeling. Accordingly, temporal changes were interpreted as descriptive patterns rather than statistically tested trends.

Laboratory findings were summarized separately by diagnostic method, sample type, and study territory. For each diagnostic method, positivity rates were calculated using the number of samples examined by that method as the corresponding denominator and were expressed as percentages. Bacteriological isolation, serological detection of F1-specific immunological markers, and real-time PCR detection of *Y. pestis* DNA were analyzed and interpreted as distinct diagnostic outcomes.

No formal inferential statistical analyses or hypothesis-testing procedures were performed. Consequently, observed differences among territories, periods, sample types, or ecological characteristics were interpreted descriptively and were not considered statistically significant associations. The epizootiological, environmental, laboratory, and spatial findings were subsequently integrated with published evidence at the interpretative stage to develop the proposed ecological framework of *Y. pestis* persistence.

## 3. Results

### 3.1. Comparative Analysis of Ecological Niches and Potential Persistence Mechanisms of Y. pestis

Published evidence indicates that the long-term persistence of *Y. pestis* may involve multiple environmental niches and bacterial adaptive states that differ substantially in the strength and type of supporting evidence (Table 2). Reported persistence ranges from short-term experimental survival to prolonged field observations, and evidence based on recovery of viable bacteria should be distinguished from molecular detection of bacterial DNA and from hypothesized ecological mechanisms.

Comparative analysis suggested that longer reported persistence tended to occur in settings providing greater environmental protection, including stable microclimatic conditions, organic substrates, intracellular localization, biofilm development, and metabolically inactive physiological states (Figure 1).

Among abiotic environments, soil and rodent burrow substrates have been most frequently associated with prolonged survival of *Y. pestis*, with viable bacteria reported under favorable environmental conditions [5,7,11,12]. Experimentally demonstrated persistence has also been reported for L-forms in nesting material for at least 427 days [17]. By contrast, evidence concerning persistence in flea carcasses and feces remains limited, and the duration of viable bacterial persistence in such organic material is uncertain (Table 2).

Several biotic environments have been investigated as potential persistence niches. Experimental studies demonstrated intracellular survival of viable *Y. pestis* in *Acanthamoeba castellanii* for at least 5–7 days [13,14,15], whereas other soil protozoa, nematodes, soil microbiota, and plant-associated habitats remain less well characterized and are supported mainly by experimental observations, ecological associations, or proposed mechanisms [2,21,22,23]. Biofilm formation and adaptive physiological states, including L-forms and persister cells, may further increase tolerance to environmental stress [16,17,18,19,20].

Thus, the available evidence supports consideration of several abiotic and biotic niches and adaptive bacterial states as potential contributors to inter-epizootic persistence. However, their evidentiary support varies considerably, and not all proposed environmental niches have been demonstrated to maintain viable *Y. pestis* under natural conditions.

### 3.2. Population Dynamics of the Great Gerbil–Flea Host–Vector System in the Natural Plague Foci of Kazakhstan

Long-term epizootiological monitoring during 2021–2025 characterized the population status of the great gerbil (*R. opimus*) and associated flea populations across the principal desert plague foci of Kazakhstan (Table 3). The annually surveyed area ranged from 737,890 to 743,890 km^2^. Mean great gerbil burrow occupancy increased descriptively from 52.9% in 2021 to 59.8% in 2025, with annual ranges of 17.61–88.55% across monitored territories. Mean great gerbil density varied from 483 to 540 individuals/km^2^, and the annual abundance category fluctuated between low and moderate.

The mean flea index on great gerbils remained relatively stable at 3.9–4.3 fleas/host. Mean flea abundance in burrows ranged from 29.4 to 53.5 fleas/burrow, while mean abundance on monitoring plots ranged from 135.5 to 173.0 fleas/plot. Estimated flea abundance increased descriptively from 15,856 individuals/km^2^ in 2021 to 27,867 individuals/km^2^ in 2025, although the annual flea abundance category remained classified as low throughout the observation period. Field observations additionally showed that flea infestation of captured rodents ranged from 0.2% to 25.7%, whereas flea occurrence in examined burrows ranged from 0.5% to 42.5%. Other rodent species were recorded at comparatively low abundance, ranging from 2.1% to 10.3% per 100 trap-nights.

Overall, the 2021–2025 surveillance data document the continued presence of great gerbil populations and associated flea populations across the monitored desert plague territories. These host–vector observations provide the ecological context for evaluating their potential contribution to the maintenance and recurrent activity of *Y. pestis* in the natural plague foci of Kazakhstan.

### 3.3. Spatial Distribution of Persistent Enzootic Cores in the Natural Plague Foci of Kazakhstan

Long-term epizootiological records from 2000–2025 combined with GIS-based spatial analysis identified geographically localized areas of recurrent plague activity within the natural plague foci of Kazakhstan. These areas, hereafter referred to as enzootic cores, were characterized by repeated detection of plague activity over the observation period and were concentrated predominantly within desert and semi-desert landscapes (Figure 2).

Four major spatial clusters of enzootic cores were identified:

– Western cluster (Ural–Emba and Ustyurt regions), 18,131 km^2^;
– Aral–Kyzylkum cluster, 27,042 km^2^;
– Balkhash cluster, 56,239 km^2^;
– Ili Intermountain cluster, 78,236 km^2^.

These clusters contained areas with recurrent *Y. pestis* detection and/or serological evidence of plague activity during long-term surveillance. Their locations also overlapped with territories in which human plague cases and outbreaks were historically recorded [33]. Fine-scale spatial analysis showed that recurrent plague activity was heterogeneously distributed within the larger natural foci. Enzootic cores occurred predominantly in areas associated with alluvial and deluvial–proluvial deposits and shallow freshwater groundwater, whereas epizootic activity was less consistently recorded in aeolian sand and more arid landscapes [33].

Descriptive temporal analysis further showed that 79.0% of infected sectors and 73.7% of *Y. pestis* isolates included in the corresponding long-term dataset were recorded during descending phases of solar cycles, with the reported frequency being 2.7-fold higher than during ascending phases [33]. These observations are presented as descriptive temporal patterns and were not subjected to inferential testing in the present study.

Overall, the spatial analysis identified persistent enzootic cores as geographically restricted areas of recurrent plague activity within the much larger natural plague territories of Kazakhstan. Their long-term spatial recurrence provides a framework for examining the ecological conditions associated with repeated detection and re-emergence of plague activity, but does not by itself demonstrate continuous persistence of viable *Y. pestis* within these locations.

### 3.4. Field and Laboratory Characterization of Enzootic Core Areas

Field investigations conducted during 2021–2023 covered approximately 120,000 km^2^ of natural plague foci in Kazakhstan. Five autonomous foci containing recurrent enzootic core areas (Ural–Emba, North Aral, Aral–Karakum, Balkhash, and Ili Intermountain) were examined together with three comparison foci (Kyzylkum, Moyynkum, and Betpakdala). A total of 4586 field samples were collected, comprising 1388 samples of rodent-burrow soil, 725 burrow-substrate samples, 399 samples of feeding-chamber contents, 1168 sets of rodent biological samples, including liver, spleen, blood, and serum, 868 ectoparasite suspensions, and 38 skeletal remains.

Laboratory examination combined bacteriological, serological, and molecular methods according to sample type (Table 4). Detailed diagnostic results according to sample type and method are presented in Supplementary Table S1.

Bacteriological examination was performed on 4548 samples and yielded 24 *Y. pestis* isolates (0.53%). Culture-positive samples were detected in the Ural–Emba (3/572; 0.52%), North Aral (1/573; 0.17%), Aral–Karakum (10/575; 1.74%), Balkhash (7/688; 1.02%), and Ili Intermountain (3/523; 0.57%) foci, whereas no culture-positive samples were detected in the Kyzylkum, Moyynkum, or Betpakdala comparison foci. All 24 recovered isolates belonged to the Medievalis biovar. Nineteen exhibited the typical phenotypic characteristics of this biovar, whereas five isolates showed atypical phenotypic features, including reduced F1 production and/or a decreased proportion of pigmented colonies.

F1-specific immunological testing was performed on 2074 samples comprising rodent sera, ectoparasite suspensions, and skeletal remains. Among the 1168 rodent serum samples, eight were positive for F1 capsular antigen, and the same eight samples were also positive for anti-F1 antibodies. F1 antigen was additionally detected in 66 ectoparasite suspensions. Overall, 74 samples (3.57%) were positive by F1-specific immunological testing. Positive findings occurred in the Ural–Emba (3/265; 1.13%), North Aral (11/265; 4.15%), Aral–Karakum (4/265; 1.51%), Balkhash (12/378; 3.17%), and Ili Intermountain (44/213; 20.66%) foci, while no positive serological results were obtained in the three comparison foci.

Real-time PCR was applied to all 4586 field samples and detected *Y. pestis*-specific DNA in 88 samples (1.92%) (Table 4). PCR-positive findings occurred exclusively in the five foci containing recurrent enzootic core areas, whereas no positive samples were detected in the three comparison foci. By sample category, PCR-positive results were obtained from burrow soil (4/1388; 0.29%), burrow substrates (6/725; 0.83%), feeding-chamber contents (4/399; 1.00%), rodent sera (8/1168; 0.68%), and ectoparasite suspensions (66/868; 7.60%); none of the 38 skeletal-remain samples was PCR-positive. The highest proportion of PCR-positive findings was observed among ectoparasite suspensions.

Taken together, culture recovery, serological findings, and molecular detection showed that laboratory evidence of *Y. pestis* circulation during 2021–2023 was concentrated in the five investigated foci containing recurrent enzootic core areas, whereas none of the three comparison foci yielded positive results by the applied methods. Importantly, the different diagnostic endpoints represent distinct levels of evidence: bacteriological isolation confirms recovery of viable *Y. pestis*, whereas serological findings and PCR detection indicate specific immunological markers and bacterial DNA, respectively, and do not independently demonstrate bacterial viability.

### 3.5. Long-Term Dynamics of the Great Gerbil Range and Environmental Patterns Associated with Plague Activity

Long-term surveillance data indicated substantial changes in the spatial distribution of the great gerbil (*R. opimus*) in the Betpakdala Desert during 1990–2025. Over this period, the area occupied by *R. opimus*, corresponding to the spatial extent of the Betpakdala autonomous plague focus, increased by 50.12% and reached approximately 60,140 km^2^. The northern distribution limit extended beyond 47° N, with new gerbil colonies recorded in areas outside the previously documented range. These observations indicate a marked northward and spatial expansion of the principal reservoir host within this natural plague focus [33].

Long-term surveillance also showed pronounced seasonal variation in plague activity. Epizootic manifestations were recorded predominantly between April and November, coinciding with the period of greatest activity of rodent hosts and flea vectors. Temporal comparison with solar-cycle phases showed that 79.0% of plague-positive sectors and 73.7% of *Y. pestis* isolates were recorded during descending phases of the analyzed solar cycles; the reported frequency of plague activity during descending phases was 2.7-fold higher than during ascending phases [33].

Together, these long-term observations demonstrate temporal and spatial variation in host distribution and plague epizootic activity in Kazakhstan. The expansion of the *R. opimus* range in Betpakdala and the concentration of plague detections during particular seasonal and longer-term periods provide descriptive evidence of the dynamic spatial and temporal organization of natural plague foci. Because these analyses were descriptive, the observed associations should not be interpreted as demonstrating a causal effect of climatic or solar-cycle variability on *Y. pestis* circulation.

### 3.6. An Integrated Ecological Model of Long-Term Y. pestis Persistence in Natural Plague Foci

Integration of the field observations, long-term surveillance data, and published evidence considered in this study provides a basis for a conceptual model of *Y. pestis* persistence in natural plague foci (Figure 3). Rather than attributing long-term maintenance to a single mechanism, the model considers interactions among the classical rodent–flea transmission cycle, spatially structured host populations, environmental substrates, and adaptive bacterial states. The strength of evidence, however, differs among these components, and several proposed environmental mechanisms remain hypothetical rather than demonstrated reservoirs of viable *Y. pestis*.

The rodent–flea system represents the best-established biological component of plague maintenance. In Kazakhstan, the spatial organization of great gerbil (*R. opimus*) populations may further contribute to long-term enzootic continuity through connectivity among colonies. Local interruption of transmission within individual colonies does not necessarily imply disappearance of the pathogen from the wider focus, where circulation may continue in other host–vector populations.

Environmental substrates may provide additional persistence niches. In the present study, *Y. pestis* was recovered by culture from several field-material categories, while real-time PCR additionally detected *Y. pestis*-specific DNA in burrow soil, burrow substrates, feeding-chamber contents, rodent sera, and ectoparasite suspensions. These findings demonstrate the presence of viable bacteria in culture-positive material and bacterial DNA in PCR-positive samples, respectively, but PCR detection alone does not establish prolonged environmental viability or an independent environmental reservoir.

Experimental and observational studies cited in this review have proposed several additional mechanisms that could contribute to environmental survival, including biofilm formation [16,19,20], interactions with free-living amoebae [13,14,15,23], L-forms and persister states [17,18], associations with plant rhizospheres [21,22], and transition to a viable-but-non-culturable (VBNC) state [9]. These mechanisms may enhance survival under particular environmental conditions, but their contribution to long-term persistence in Kazakhstan’s natural plague foci has not been directly quantified in the present study.

Molecular-genetic evidence provides a complementary perspective. Molecular typing and genomic studies have demonstrated substantial phylogenetic and geographic structuring within *Y. pestis* populations, including Central Asian lineages [34,35,36,37]. Such genetic structuring is compatible with long-term maintenance within established natural foci, although it does not identify the ecological compartment or mechanism responsible for survival during inter-epizootic intervals.

Taken together, these observations support an integrated framework in which long-term plague maintenance may emerge from interactions among established host–vector transmission, spatial connectivity of reservoir populations, environmental exposure pathways, and bacterial survival strategies. The model therefore distinguishes well-established components of plague ecology from potential persistence mechanisms requiring further experimental validation. This distinction is particularly important for surveillance: conventional monitoring of rodents and flea vectors remains central, whereas environmental and molecular investigations can provide complementary evidence and help identify mechanisms that warrant targeted investigation.

### 3.7. Study Limitations and Future Research Directions

Several limitations should be considered when interpreting the findings of this study. First, the study integrates heterogeneous evidence derived from long-term surveillance, targeted field investigations, laboratory analyses, and previously published experimental and molecular studies. These data streams differed in temporal coverage, spatial resolution, sampling design, and analytical approach and were therefore integrated conceptually rather than subjected to pooled statistical analysis.

Second, the field and laboratory data provide different levels of evidence for *Y. pestis* occurrence and persistence. Culture-positive samples demonstrate recovery of viable bacteria, whereas real-time PCR confirms the presence of *Y. pestis*-specific DNA but does not establish bacterial viability or the duration of environmental persistence. Similarly, detection of *Y. pestis* in environmental material does not by itself demonstrate that soil or other environmental substrates function as independent long-term reservoirs. Several mechanisms incorporated into the proposed model—including interactions with free-living amoebae, biofilm-associated survival, L-forms, persister cells, rhizosphere associations, and the viable-but-non-culturable state—are supported primarily by previously published experimental or geographically restricted studies and were not directly demonstrated in the present field investigation.

Third, the long-term ecological and spatial analyses were primarily descriptive. Differences in surveillance intensity, sampling effort, and temporal coverage may influence comparisons among periods and territories, and the observed associations with climatic, seasonal, and solar-cycle variables should not be interpreted as evidence of causality. Likewise, the delineation of persistent enzootic cores identifies areas of recurrent plague activity but does not demonstrate continuous presence of viable *Y. pestis* throughout the entire inter-epizootic period.

Future studies should therefore combine longitudinal standardized sampling with culture-based detection, quantitative molecular methods, genomic and metagenomic approaches, and high-resolution spatial and environmental data. Particular attention should be given to experimentally testing the viability and duration of *Y. pestis* persistence in environmental substrates and to distinguishing transient contamination from biologically relevant persistence niches. Integration of these approaches with genomic surveillance and host–vector monitoring could provide a more rigorous basis for evaluating the relative contribution of different persistence mechanisms and for refining ecological models of natural plague foci.

## 4. Discussion

The present study integrates long-term surveillance, spatial analyses, field and laboratory investigations, and published evidence to examine ecological processes associated with the long-term maintenance of plague in Kazakhstan. The recurrent concentration of plague activity within geographically defined enzootic cores, together with host–vector dynamics and laboratory evidence of *Y. pestis* circulation, supports a multifactorial interpretation of plague persistence rather than attribution to a single ecological mechanism.

The classical rodent–flea transmission cycle remains the best-established mechanism of plague maintenance and transmission during active epizootics [31,38]. Fleas may also contribute to persistence during periods of reduced transmission activity [29]. In Kazakhstan, however, plague activity is spatially heterogeneous within natural foci [33]. Metapopulation dynamics of great gerbil (*R. opimus*) colonies may contribute to landscape-scale continuity despite local extinction or temporary interruption of infection [30]. Comparable long-term spatial structuring of plague activity has been described in other endemic systems [39,40].

Environmental conditions may further influence the spatial and temporal organization of plague activity. Associations with precipitation, climatic variability, and other environmental factors have been reported in geographically distinct plague-endemic settings [41,42,43]. Soil salinity and aridity have also been associated with the distribution of plague foci, while the environmental tolerance of *Y. pestis* has been investigated as a possible contributor to persistence under particular conditions [44,45]. Nevertheless, environmental detection should not be equated with an independent reservoir: in the present study, culture-positive material demonstrated viable *Y. pestis*, whereas PCR-positive samples demonstrated bacterial DNA without establishing viability or duration of environmental persistence.

Molecular evidence provides a complementary perspective on long-term continuity. Molecular typing and genomic approaches have demonstrated substantial phylogenetic and geographical structuring within *Y. pestis* populations [34,35]. Studies of Central Asian lineages further demonstrate regional diversification and spatial structuring of plague populations [36,37]. Such genetic continuity is compatible with long-term maintenance within established natural foci but does not identify the ecological compartment in which viable bacteria persist during inter-epizootic periods.

The broader geographical literature similarly demonstrates that plague systems can remain active or re-emerge after prolonged intervals. Long-term persistence in Madagascar reflects interactions among hosts, flea vectors, and local environmental conditions [46,47]. Plague reappeared in Algeria after approximately five decades and in Libya after a prolonged period without reported cases [48,49]. Persistent plague-endemic landscapes have also been described in Brazil and elsewhere in Latin America [50,51]. These observations demonstrate recurrent plague activity across diverse ecological settings but do not imply a common mechanism of persistence among geographically distinct foci.

Additional bacterial survival mechanisms may contribute under particular environmental conditions. Persister states represent a recognized form of phenotypic bacterial persistence [32], while free-living amoebae can provide intracellular niches for pathogenic microorganisms [52]. Biofilm formation and other adaptive traits have also been proposed as mechanisms facilitating *Y. pestis* circulation and maintenance in natural-focus ecosystems [53]. In the framework proposed here, these processes should therefore be considered potential complementary mechanisms rather than established components operating simultaneously within Kazakhstan’s plague foci.

Overall, the findings support an evidence-weighted ecological framework in which established rodent–flea transmission operates within spatially structured host populations and heterogeneous environments, while additional environmental and bacterial survival mechanisms remain plausible but incompletely validated. Longitudinal studies combining standardized field sampling, culture-based detection, genomic surveillance, and environmental data will be required to determine their relative contributions to long-term plague maintenance.

## 5. Conclusions

This study integrates long-term epizootiological surveillance, spatial analyses, field and laboratory investigations, and published evidence to provide an ecological framework for understanding the long-term maintenance of *Y. pestis* in the natural plague foci of Kazakhstan. The findings indicate that plague persistence is spatially heterogeneous and is associated with recurrent enzootic core areas, the host–vector system, and the spatial organization of the great gerbil (*R. opimus*) populations.

Culture-confirmed recovery of viable *Y. pestis*, together with serological and molecular evidence of pathogen circulation, supports the continued activity of established natural plague foci. However, detection of *Y. pestis* DNA in environmental material does not by itself demonstrate bacterial viability or confirm an independent environmental reservoir. Accordingly, environmental substrates and proposed adaptive mechanisms, including biofilms, interactions with free-living amoebae, persister and VBNC states, and rhizosphere associations, should be regarded as potential complementary persistence mechanisms requiring further field and experimental validation.

Overall, the proposed model emphasizes interactions among established host–vector transmission, spatially structured reservoir populations, environmental conditions, and potential bacterial survival strategies. This evidence-weighted framework may support more targeted ecological surveillance and future studies aimed at identifying the mechanisms underlying long-term plague persistence.

## Figures and Tables

**Figure 1:**
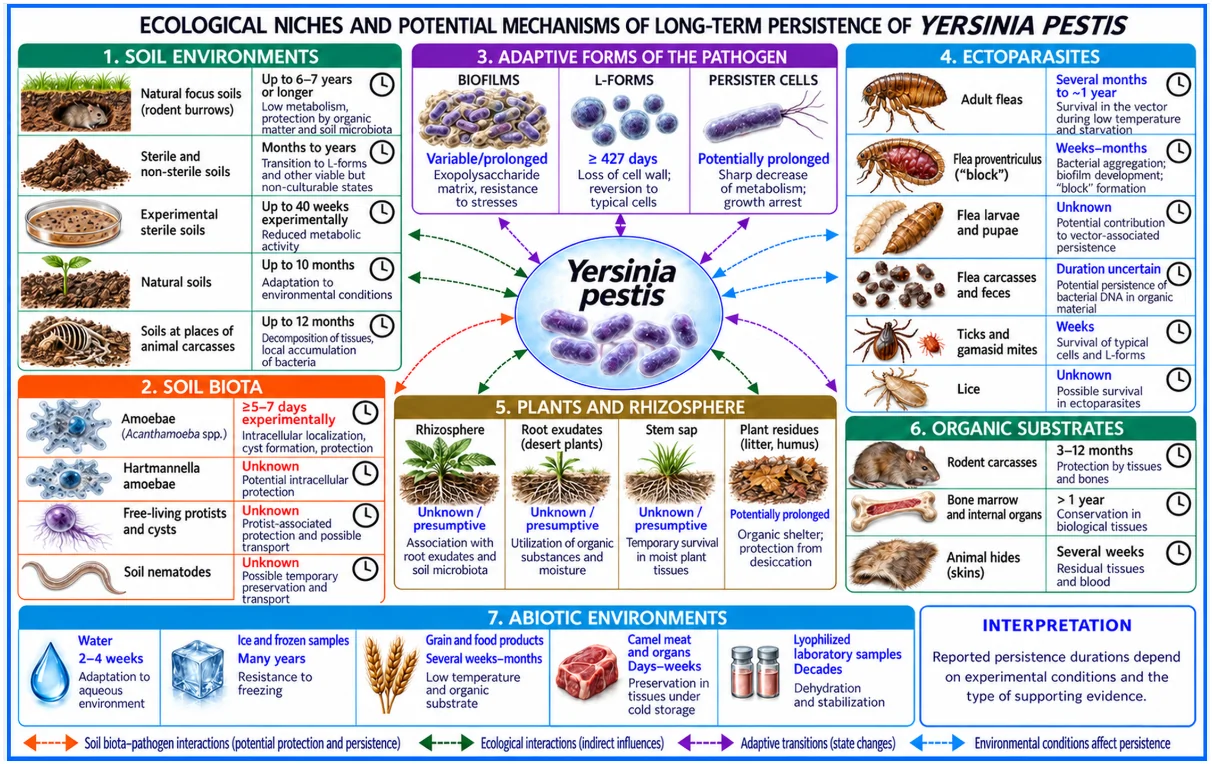
Ecological niches and mechanisms potentially contributing to the long-term persistence of Yersinia pestis in natural plague foci. The schematic synthesizes evidence reported in references [1,2,5,7,9,13,14,15,16,17,18,19,20,21,22,23,29,30,31,32]; details of individual mechanisms and their evidence levels are provided in Table 2. Note: The experimentally observed persistence duration in free-living amoebae is 5–7 days.

**Figure 2:**
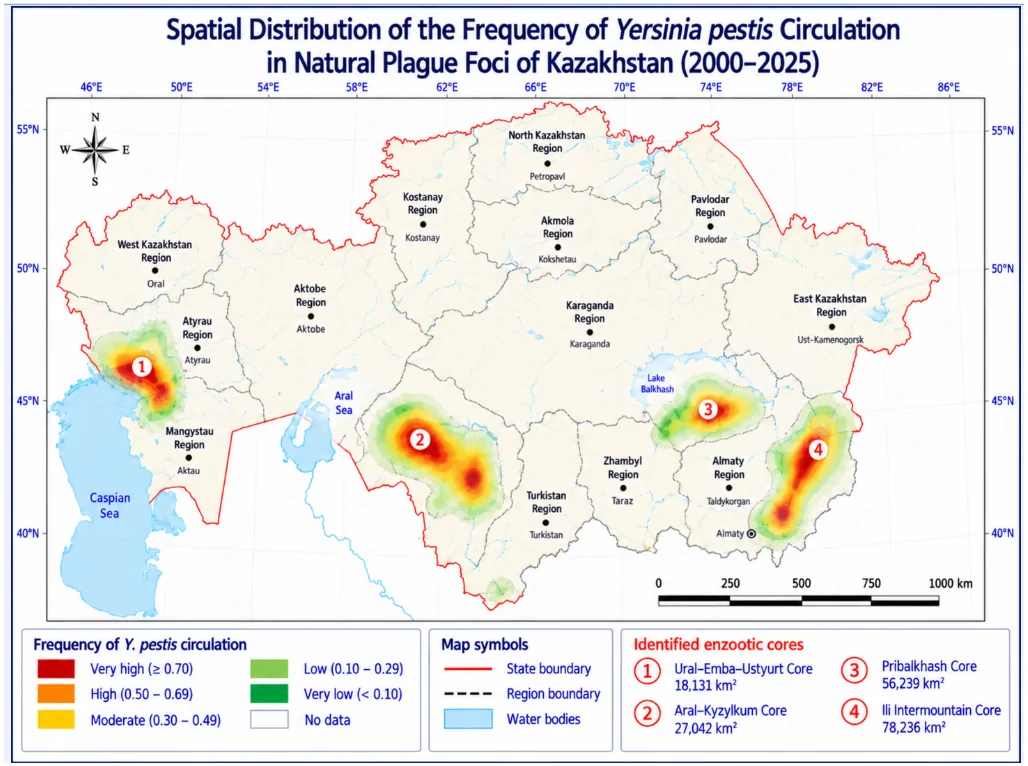
Spatial distribution of enzootic cores of *Yersinia pestis* in the natural plague foci of Kazakhstan. The map was created by the authors from long-term epizootiological surveillance and spatial data. Spatial analysis and mapping were performed using ArcGIS 10.8; the figure was subsequently prepared for publication using Mind the Graph (https://mindthegraph.com) and Adobe Illustrator (Adobe Inc., San Jose, CA, USA; https://www.adobe.com/products/illustrator.html, accessed on 13 October 2026). No third-party copyrighted cartographic material was used.

**Figure 3:**
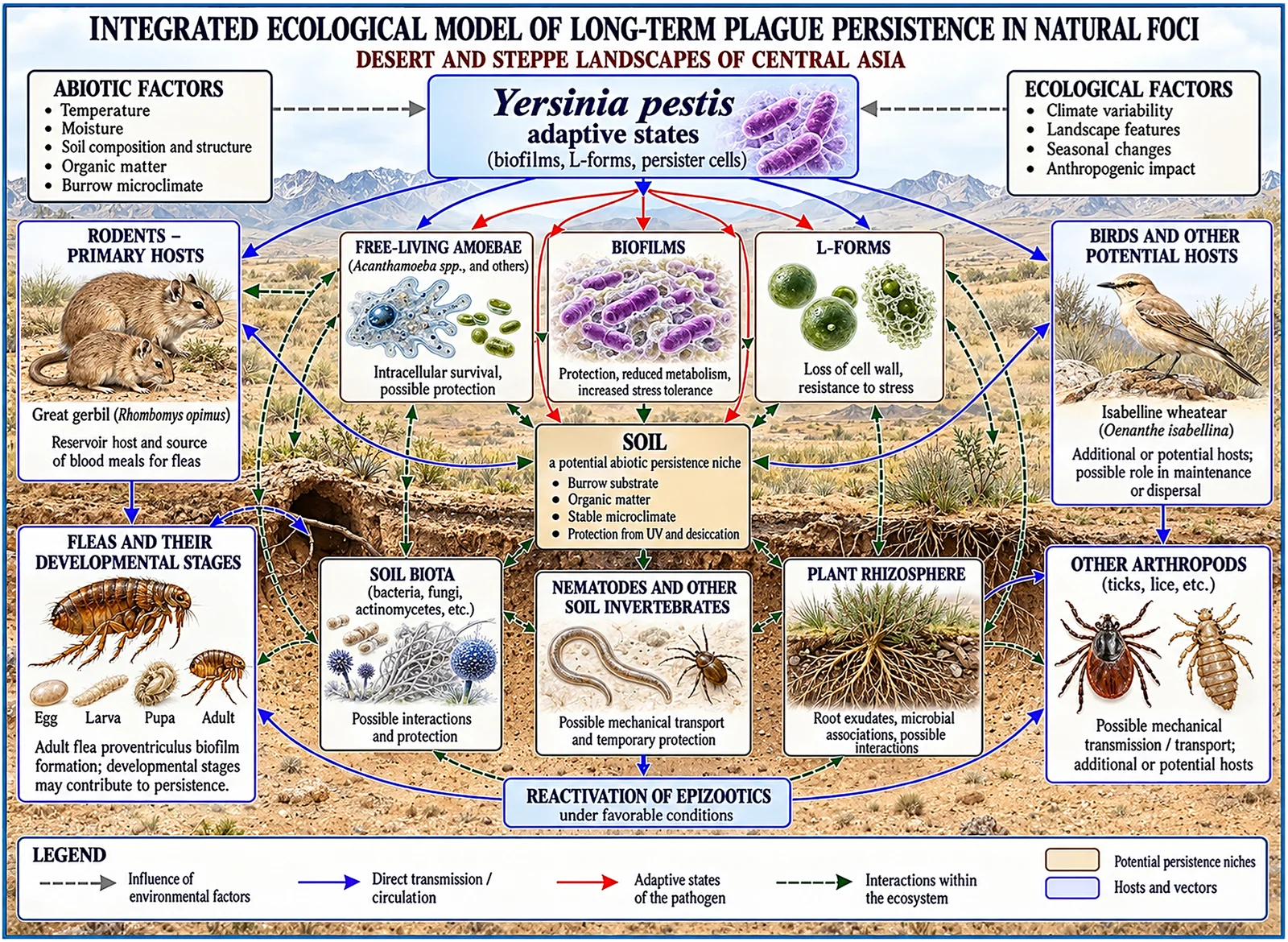
Proposed integrated ecological model of mechanisms potentially contributing to the long-term persistence of *Yersinia pestis* in natural plague foci. Solid components represent established elements of the rodent–flea transmission system, whereas proposed environmental niches and adaptive bacterial states represent potential mechanisms whose contribution may vary according to ecological context and level of experimental evidence.

**Table 1:** Summary of datasets and evidence sources used in the study.

Evidence Component	Period	Geographic Scope	Data/Material	Sample Size or Coverage	Primary Analysis	Evidence Source/Status
Historical Epizootiological Records	1926–2022	Natural plague foci of Kazakhstan	Archival records of plague activity	Long-term archival dataset	Retrospective descriptive analysis	Historical surveillance records
Long-Term Epizootiological Monitoring	1990–2025	Major natural plague territories of Kazakhstan	Reservoir-host, flea-vector, spatial, and epizootiological monitoring data	Annual surveillance records	Descriptive temporal and spatial analysis	National surveillance data
Original Field And Laboratory Investigations	2021–2023	Selected enzootic core areas and comparison territories	Environmental and biological samples	4586 samples; approximately 120,000 km^2^ surveyed	Bacteriological, serological, molecular, environmental, and spatial analyses	Original data generated in the present study
Recent Host–Vector Monitoring	2021–2025	Natural plague territories of Kazakhstan	*Rhombomys opimus* population and flea-vector indicators	Approximately 738,000–744,000 km^2^ surveyed annually	Descriptive analysis of annual host–vector indicators	National surveillance data
Published Ecological Evidence	Various	Kazakhstan and other plague-endemic regions	Published experimental, field, and ecological studies	Not applicable	Comparative ecological synthesis	Previously published evidence
Integrated Ecological Framework	Present study	Natural plague foci of Kazakhstan	Synthesis of the above evidence streams	Not applicable	Integrative interpretation	Conceptual synthesis developed in the present study

**Table 2:** Ecological niches and mechanisms potentially contributing to the persistence of *Yersinia pestis* in natural plague foci.

Ecological Niche, Substrate, Host/Vector, or Physiological State	Reported Persistence	Proposed Mechanism	Evidence and Potential Ecological Significance	Representative References
Abiotic environmental niches				
Surface and deeper soil layers	6–7 years or longer, as reported	Stable temperature and moisture; reduced UV exposure; organic matter; reduced metabolic activity	Viable *Y. pestis* reported under favorable conditions; potential long-term environmental persistence niche	Rivkus & Blummer, 2016 [2]; Larina et al., 1989 [5]; Ayyadurai et al., 2008 [7]
Rodent burrow substrates and nest material	Several years (reported); ≥427 days for L-forms under experimental conditions	Protected microclimate, moisture, organic material, and reduced environmental exposure	Potential protected persistence niche; viability demonstrated under specific experimental/field conditions	Rivkus & Blummer, 2016 [2]; Larina et al., 1989 [5]; Bazanova et al., 1997 [17]
Organic debris and soil organic matter	Duration uncertain; potentially months to years	Physical protection, nutrient availability, binding to organic compounds and colloids	Potential temporary environmental persistence niche	Pollitzer, 1954 [1]; Rivkus & Blummer, 2016 [2]
Desert soil microhabitats, sand, and aeolian particles	Unknown	Reduced UV exposure, microclimatic buffering, association with fine particles	Hypothesized environmental persistence and dispersal niche; viability not established	Rivkus & Blummer, 2016 [2]
Water, moist soil, and mud	Weeks to months	Low temperature, moisture, organic content, and protection from desiccation	Short-term environmental survival reported; ecological role remains uncertain	Ayyadurai et al., 2008 [7]; Pawlowski et al., 2011 [9]
Biotic niches and host–vector components				
Free-living amoebae (*Acanthamoeba* spp.)	5–7 days experimentally	Intracellular survival and protection	Experimentally demonstrated intracellular viability; potential environmental persistence niche	Markman et al., 2018 [13]; Benavides-Montaño & Vadyvaloo, 2017 [14]
Other free-living amoebae and soil protozoa	Unknown	Potential intracellular protection	Hypothesized auxiliary persistence niche; limited direct evidence	Koshel et al., 2015 [23]; Greub & Raoult, 2004 [15]
Soil microbiota	Unknown	Community interactions, nutrient exchange, and bioprotection	Potential ecological buffering; direct persistence role remains uncertain	Larina et al., 1989 [5]; Rivkus & Blummer, 1986 [22]; Rivkus & Blummer, 2016 [2]
Nematodes and other soil invertebrates	Unknown	Passive transport, transient carriage, and protection	Potential transient carriers; reservoir function not established	Pollitzer, 1954 [1]; Rivkus & Blummer, 2016 [2]
Rhizosphere and other plant-associated habitats	Unknown	Root exudates, microenvironment modification, adherence to plant material	Hypothesized additional ecological niche; evidence mainly ecological/experimental	Rivkus et al., 1993 [21]; Rivkus & Blummer, 2016 [2]
Flea larvae and pupae	Unknown	Maintenance within flea populations	Potential contribution to vector-associated persistence; evidence limited	Pollitzer, 1954 [1]; Wimsatt & Biggins, 2009 [29]
Adult fleas	Several months to ~1 year	Proventricular biofilm formation and prolonged infection	Established vector and transmission component capable of prolonged pathogen carriage	Pollitzer, 1954 [1]; Jarrett et al., 2004 [16]; Wimsatt & Biggins, 2009 [29]
Flea carcasses and feces	Duration uncertain	Protection within biological and organic material may prolong bacterial or DNA persistence	Potential transient source of environmental contamination; direct evidence for prolonged viable bacterial persistence remains limited	Wimsatt & Biggins, 2009 [29]
Great gerbil (*Rhombomys opimus*)	Population-level persistence	Maintenance of flea populations and host–vector transmission cycles	Principal vertebrate reservoir host in desert plague foci	Schmid et al., 2012 [30]
Other rodents	Population-level persistence	Maintenance of flea populations and pathogen transmission cycles	Additional vertebrate reservoir hosts; contribution varies among plague foci	Pollitzer, 1954 [1]; Gage & Kosoy, 2005 [31]; Rivkus & Blummer, 2016 [2]
Adaptive physiological states of *Yersinia pestis*				
Biofilms	Variable; potentially prolonged	Extracellular polymeric matrix protects bacterial cells from environmental stress	Experimentally supported mechanism increasing stress tolerance and vector-associated persistence	Jarrett et al., 2004 [16]; Konnov et al., 2009 [19]; Bazanova et al., 2018 [20]
L-forms	≥427 days experimentally	Cell-wall deficiency and reduced metabolic activity	Experimentally demonstrated prolonged survival under specific conditions	Bazanova et al., 1997 [17]
Persister and other low-metabolic states	Potentially prolonged	Reversible metabolic dormancy, starvation response, and stress tolerance	Potential mechanism of survival during unfavorable conditions	Lewis, 2007 [18]; Balaban et al., 2019 [32]
VBNC and stress-adapted states	Unknown; potentially prolonged	Reduced culturability and physiological adaptation to environmental stress	Proposed explanation for persistence without conventional bacterial isolation; ecological role remains uncertain	Pawlowski et al., 2011 [9]

Note: The strength of evidence supporting the listed niches and mechanisms varies, ranging from recovery of viable bacteria and experimentally demonstrated survival to molecular detection of *Y. pestis* DNA, ecological associations, and hypothesized mechanisms. Detection of bacterial DNA alone does not demonstrate bacterial viability or establish an environmental reservoir. Reported persistence periods should therefore be interpreted according to the type of evidence specified for each entry.

**Table 3:** Population dynamics of the great gerbil (*Rhombomys opimus*) and flea abundance in the natural plague foci of Kazakhstan, 2021–2025.

Parameter	2021	2022	2023	2024	2025
Surveyed area (km^2^)	741,650	738,450	742,450	743,890	737,890
Great gerbil burrow occupancy (%) (range)	17.61–88.30	20.45–86.20	26.30–86.12	30.70–88.23	31.20–88.55
Great gerbil burrow occupancy (%) (mean)	52.9	53.3	56.2	59.5	59.8
Great gerbil density (individuals/km^2^) (range)	52–1028	49–916	71–991	94–951	68–914
Great gerbil density (individuals/km^2^) (mean)	540	483	531	523	491
Great gerbil abundance level	Moderate	Low	Moderate	Moderate	Low
Mean flea index on great gerbils (fleas/host)	4.0	4.0	3.9	4.3	4.3
Mean flea abundance in burrows (fleas/burrow)	34.0	53.5	30.5	29.4	42.5
Mean flea abundance (fleas/plot)	138.0	150.0	173.0	135.5	155.7
Estimated flea abundance (individuals/km^2^)	15,856	16,890	25,729	27,366	27,867
Flea abundance level	Low	Low	Low	Low	Low

Note: km^2^, square kilometer. Population status categories were assigned according to the official epizootiological monitoring criteria adopted by the M. Aikimbayev National Scientific Center for Especially Dangerous Infections, Republic of Kazakhstan.

**Table 4:** Bacteriological, F1-specific immunological, and real-time PCR results for field samples collected from eight autonomous natural plague foci of Kazakhstan, 2021–2023.

Autonomous Plague Focus	Bacteriology Tested	Positive, *n* (%)	F1 Immunological Testing, *n*	Positive, *n* (%)	Real-Time PCR Tested	Positive, *n* (%)
Ural–Emba	572	3 (0.52)	265	3 (1.13)	577	4 (0.69)
North Aral	573	1 (0.17)	265	11 (4.15)	578	14 (2.42)
Aral–Karakum	575	10 (1.74)	265	4 (1.51)	580	7 (1.21)
Balkhash	688	7 (1.02)	378	12 (3.17)	693	15 (2.16)
Ili Intermountain	523	3 (0.57)	213	44 (20.66)	528	48 (9.09)
Kyzylkum	589	0 (0.00)	278	0 (0.00)	593	0 (0.00)
Moyynkum	544	0 (0.00)	235	0 (0.00)	549	0 (0.00)
Betpakdala	484	0 (0.00)	175	0 (0.00)	488	0 (0.00)
Total	4548	24 (0.53)	2074	74 (3.57)	4586	88 (1.92)

Note: The number of samples examined differed among diagnostic methods because diagnostic testing was performed according to sample type. Bacteriological examination included burrow soil, burrow substrates, feeding-chamber contents, rodent biological materials (liver, spleen, and blood), and ectoparasite suspensions (*n* = 4548); F1-specific immunological testing included rodent sera, ectoparasite suspensions, and skeletal remains (*n* = 2074); and real-time PCR was applied to all six sample categories (*n* = 4586). Culture positivity indicates recovery of viable *Y. pestis*, whereas serological and PCR-positive findings represent detection of the corresponding immunological markers and *Y. pestis*-specific DNA, respectively, and do not independently demonstrate bacterial viability.

## Data Availability

The data presented in this study are available from the corresponding author upon reasonable request. The data are not publicly available due to institutional and national restrictions related to epidemiological surveillance of particularly dangerous infections.

## Supplementary Materials

The following supporting information can be downloaded at https://www.mdpi.com/article/10.3390/pathogens15101091/s1, Table S1: Diagnostic testing of samples collected during plague surveillance and recovery of viable *Yersinia pestis* isolates.
