# Supplementary material for: Ecological Mechanisms Underlying the Persistence of Yersinia pestis in the Natural Plague Foci of Kazakhstan: An Integrated Ecological Framework
Source: Pathogens. 2026 Oct 20;15(10):1091. doi: 10.3390/pathogens15101091 (PMC13708159; doi:10.3390/pathogens15101091)
Supplement: Supplementary file 1 [file pathogens-15-01091-s001.zip › pathogens-4560944-supplementary.pdf]

Supplementary Table S1. Diagnostic testing of samples collected during plague surveillance and recovery of viable *Yersinia pestis* isolates

Sample type	Samples tested by bacteriology, *n*	Bacteriology-positive samples / viable *Y. pestis* isolates, *n*	Samples tested by F1-specific immunological assays, *n*	F1-specific positive samples, *n*	Samples tested by real-time PCR, *n*	Real-time PCR-positive samples, *n*
Soil from rodent burrows	1388	0	NA	NA	1388	4
Substrates from rodent burrows	725	0	NA	NA	725	6
Contents of food-storage chambers in rodent burrows	399	0	NA	NA	399	4
Rodent biological samples*	1168	8	1168	8**	1168	8
Ectoparasite suspensions	868	16	868	66***	868	66
Rodent skeletal remains	NA	NA	38	0	38	0
**Total**	**4548**	**24**	**2074**	**74**	**4586**	**88**

Notes:
NA, not applicable/not tested; F1, fraction 1 capsular antigen of Yersinia pestis; PCR, polymerase chain reaction.

* For bacteriological examination of rodents, liver, spleen, and blood samples were used; serum samples were used for F1-specific immunological testing.

** Eight rodent serum samples were positive for both F1 capsular antigen and anti-F1 antibodies; the same eight samples were positive in both assays.

*** F1 capsular antigen was detected in 66 ectoparasite suspensions.
